# Supplementary material for: Proteomic, Metabolomic, and Lipidomic Analyses of Lung Tissue Exposed to Mustard Gas
Source: Metabolites. 2022 Aug 30;12(9):815. doi: 10.3390/metabo12090815 (PMC9501011; doi:10.3390/metabo12090815)
Supplement: Supplementary file 1 [file metabolites-12-00815-s001.zip › Supplemental Figures for PROTEOMIC, METABOLOMIC, and LIPIDOMIC.pptx]

## Slide 1
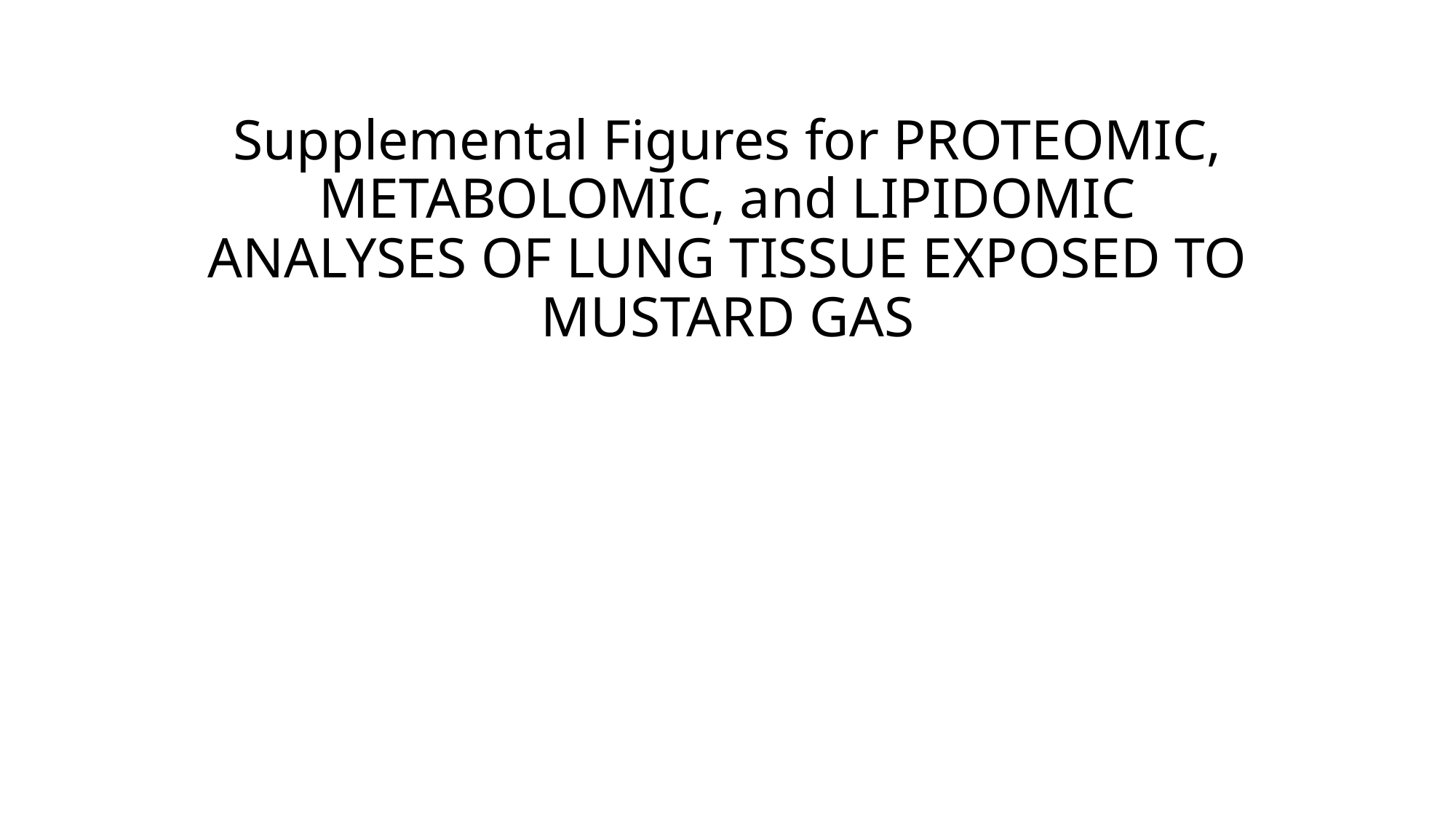

# Supplemental Figures for PROTEOMIC, METABOLOMIC, and LIPIDOMIC ANALYSES OF LUNG TISSUE EXPOSED TO MUSTARD GAS

## Slide 2
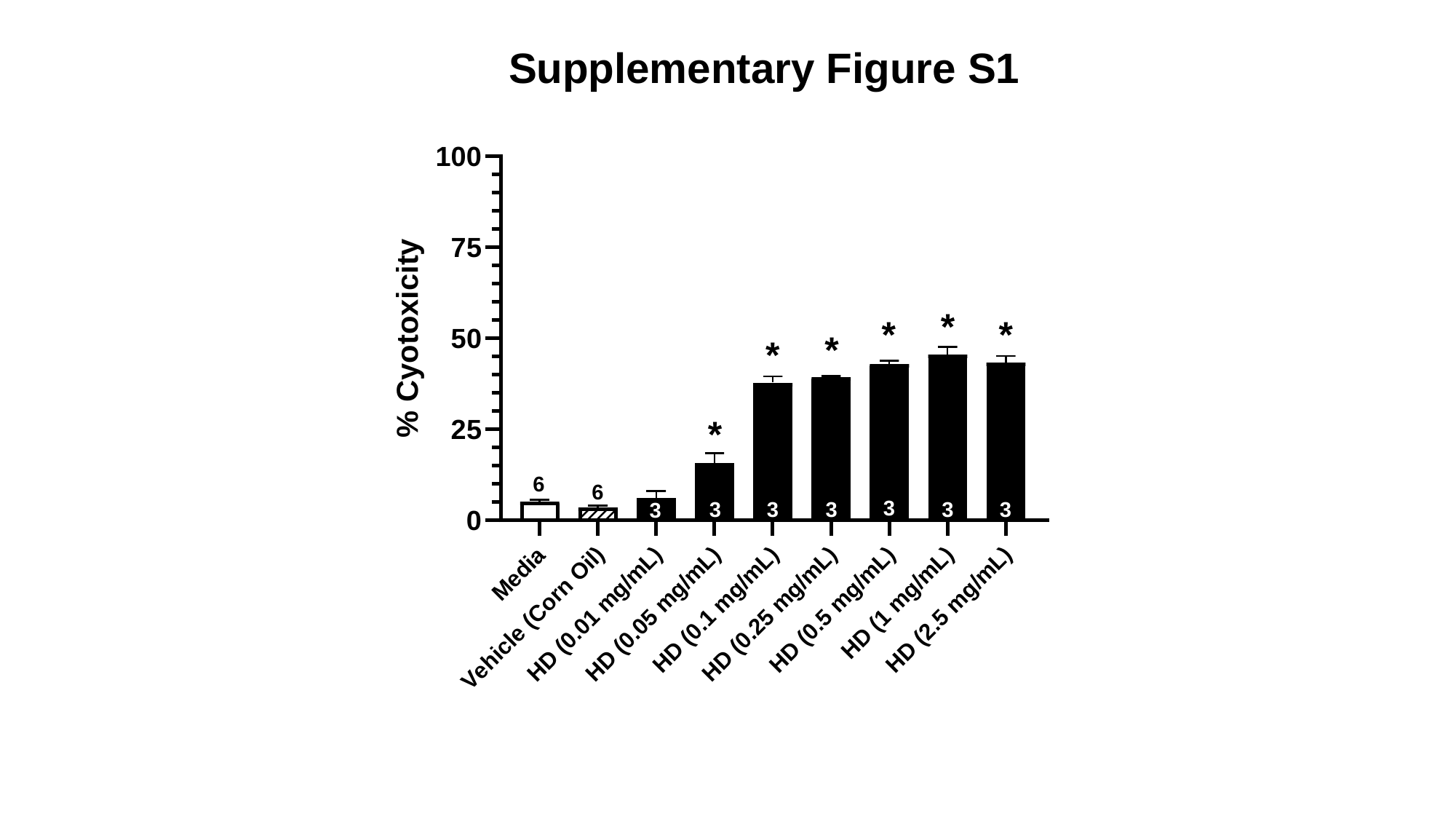

Supplementary Figure S1
*
*
*
*
*
*
6
6
3
3
3
3
3
3
3

## Slide 3
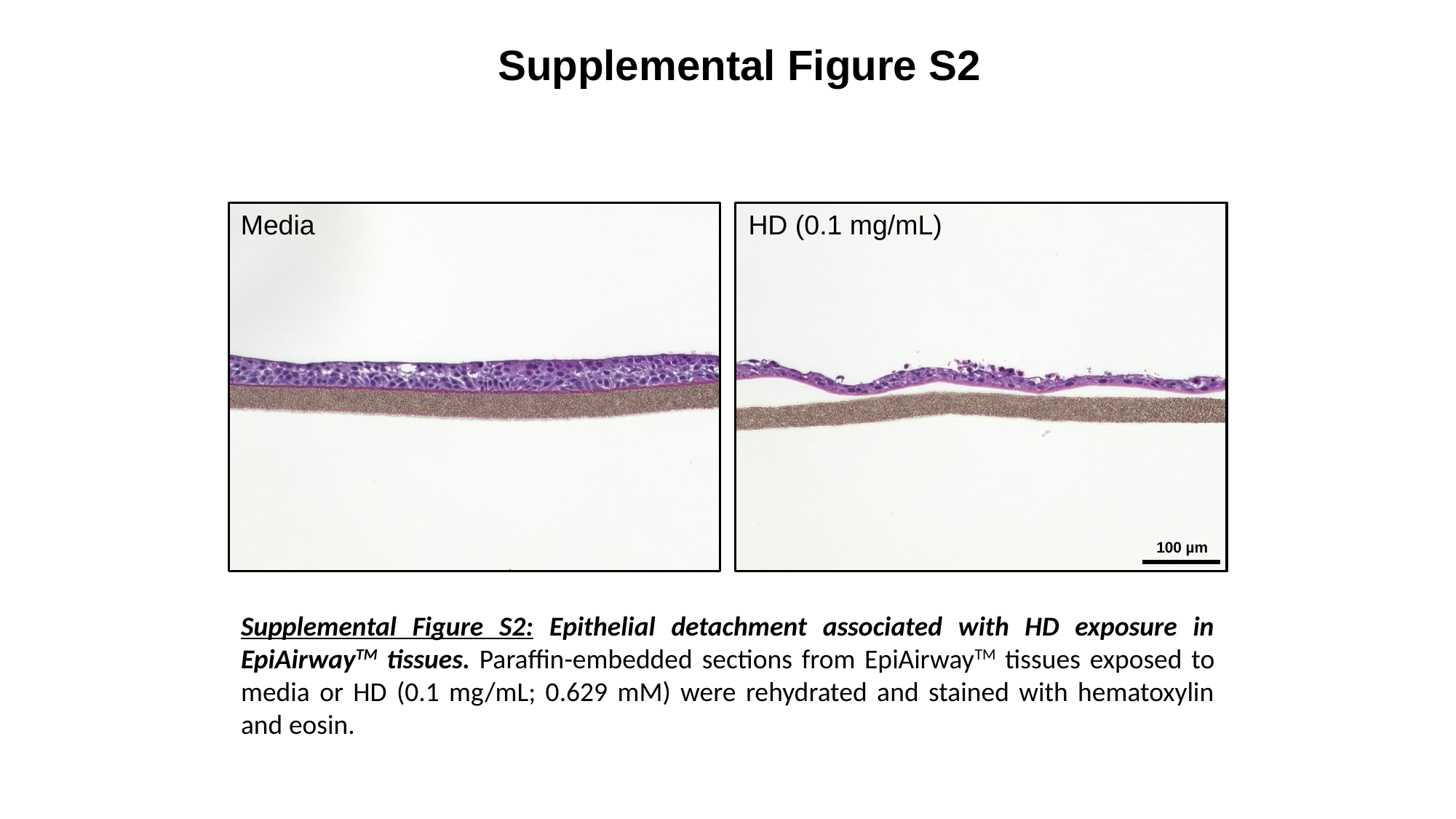

Supplemental Figure S2
HD (0.1 mg/mL)
Media
100 µm
Supplemental Figure S2: Epithelial detachment associated with HD exposure in EpiAirwayTM tissues. Paraffin-embedded sections from EpiAirwayTM tissues exposed to media or HD (0.1 mg/mL; 0.629 mM) were rehydrated and stained with hematoxylin and eosin.

## Slide 4
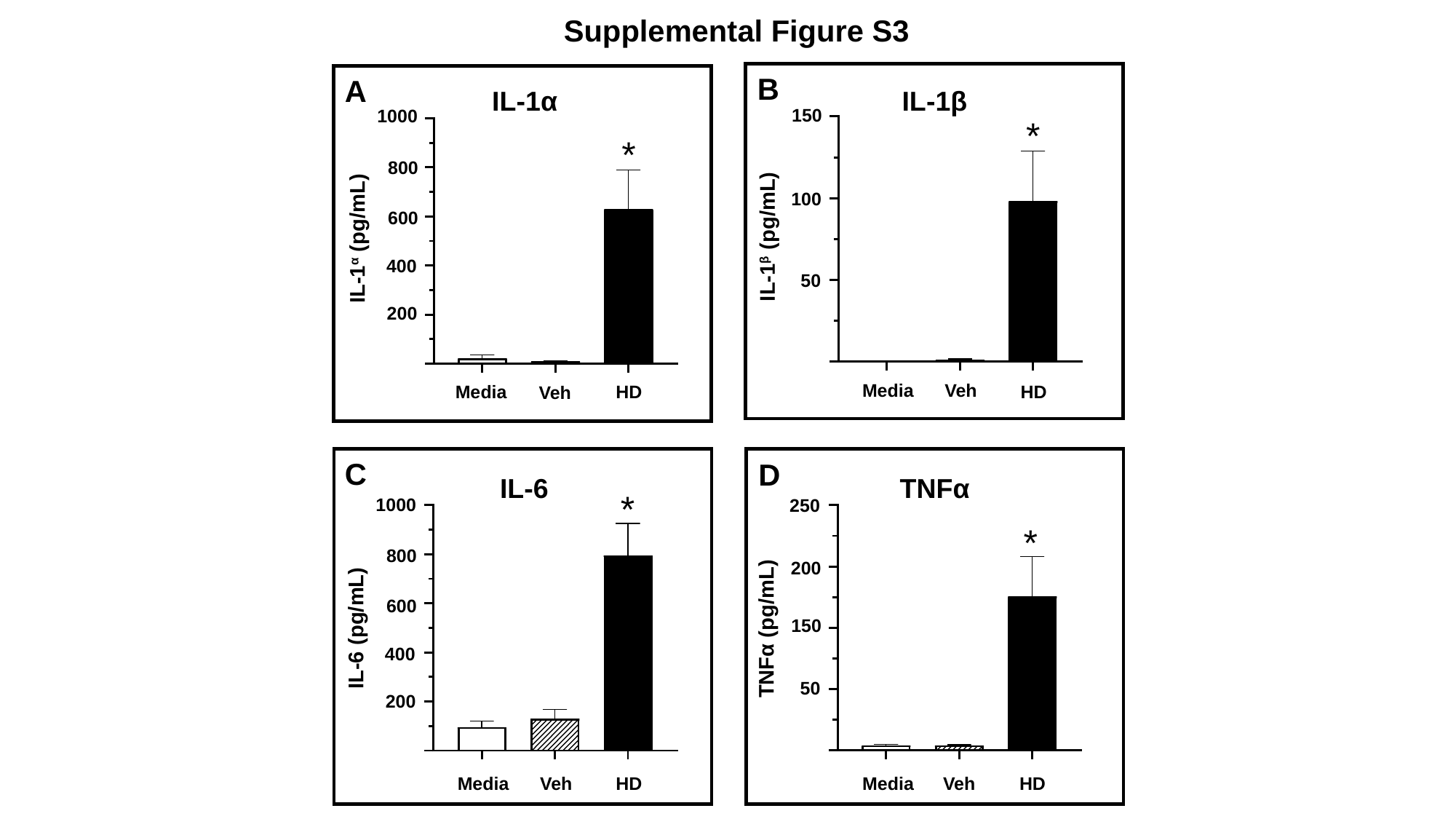

Supplemental Figure S3
B
A
IL-1β
150
*
100
IL-1β (pg/mL)
50
Media
Veh
HD
IL-1α
1000
*
800
600
IL-1α (pg/mL)
400
200
HD
Media
Veh
C
D
TNFα
IL-6
*
1000
250
*
800
200
600
150
IL-6 (pg/mL)
TNFα (pg/mL)
400
50
200
HD
Media
Media
Veh
HD
Veh

## Slide 5
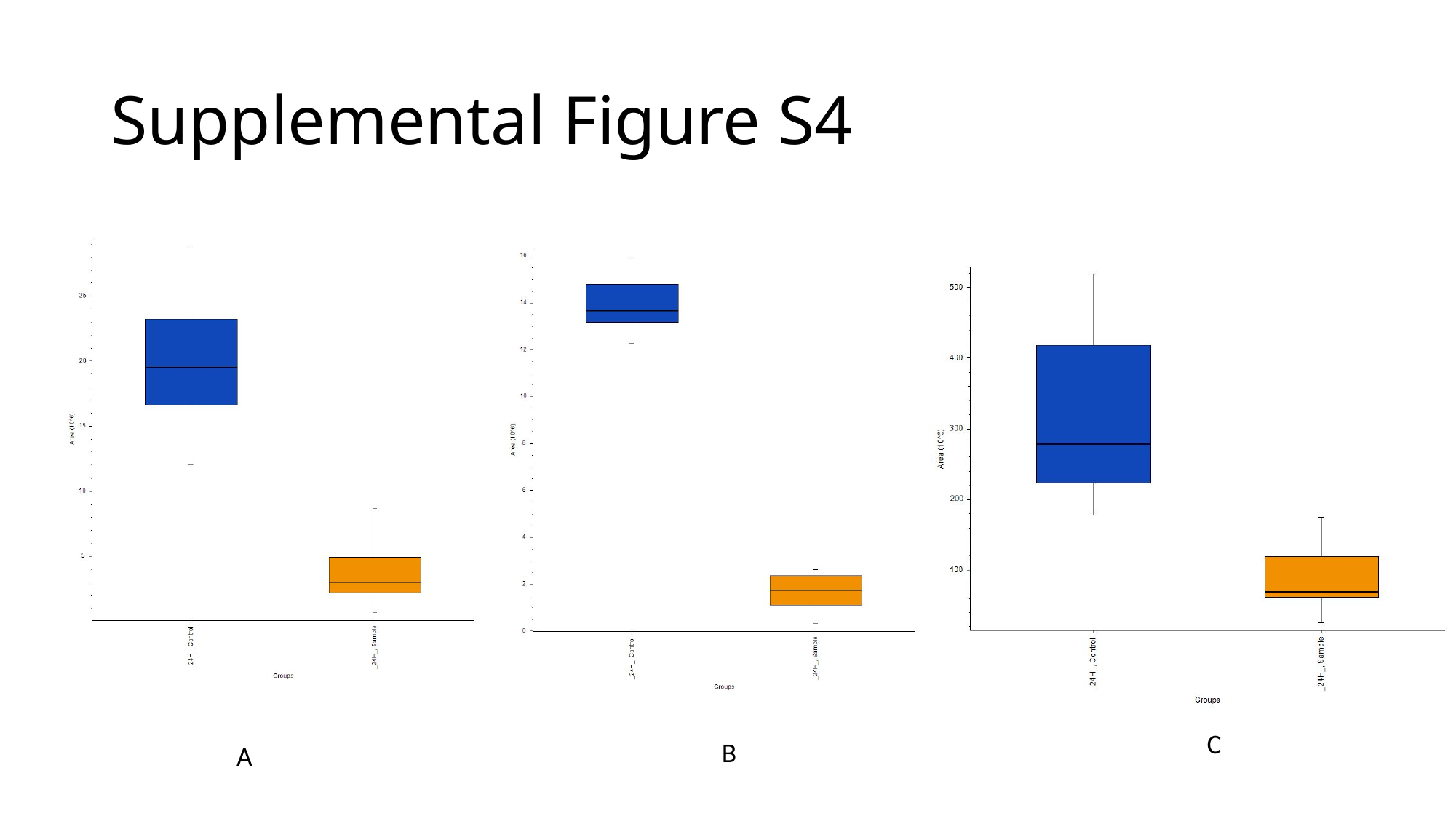

# Supplemental Figure S4
C
B
A
